# Smart Tattoo Sensors 2.0: A Ten-Year Progress Report through a Narrative Review

**DOI:** 10.3390/bioengineering11040376

**Published:** 2024-04-13

**Authors:** Antonia Pirrera, Daniele Giansanti

**Affiliations:** Centro Nazionale TISP, Istituto Superiore di Sanità, Viale Regina Elena 299, 00161 Roma, Italy

**Keywords:** tattoo, biosensor, biosensing, skin sensor

## Abstract

The increased interest in sensing tattoos reflects a shift in wearable technology, emphasizing their flexible, skin-adherent nature. These devices, driven by advancements in nanotechnology and materials science, offer highly sensitive and customizable sensors. The growing body of research in this area indicates a rising curiosity in their design and applications, with potential uses ranging from vital sign monitoring to biomarker detection. Sensing tattoos present a promising avenue in wearable healthcare technology, attracting attention from researchers, clinicians, and technology enthusiasts. The objective of this study is to analyze the development, application, and integration of the sensing tattoos in the health domain. A review was conducted on PubMed and Scopus, applying a standard checklist and a qualification process. The outcome reported 37 studies. Sensing tattoos hold transformative potential in health monitoring and physiological sensing, driven by their focus on affordability, user-friendly design, and versatile sensorization solutions. Despite their promise, ongoing refinement is essential, addressing limitations in adhesion, signal quality, biocompatibility, and regulatory complexities. Identified opportunities, including non-invasive health monitoring, multiplexed detection, and cost-effective fabrication methods, open avenues for personalized healthcare applications. However, bridging gaps in medical device standards, cybersecurity, and regulatory compliance is imperative for seamless integration. A key theme calls for a holistic, user-centric approach, emphasizing interdisciplinary collaboration. Balancing innovation with practicality, prioritizing ethics, and fostering collaboration are crucial for the evolution of these technologies. The dynamic state of the field is evident, with active exploration of new frontiers. This overview also provides a roadmap, urging scholars, industry players, and regulators to collectively contribute to the responsible integration of sensing tattoos into daily life.

## 1. Introduction

Tattoos, tracing back to ancient rituals, have evolved into a widespread practice with diverse cultural and personal meanings. Notably, the Tyrolean Iceman (Ötzi) bore 61 tattoos, suggesting potential therapeutic functions [1]. Recent surveys show a substantial increase in tattoo prevalence, with 12% of Europeans and 24% of U.S. citizens reported to have tattoos [2,3]. In Italy, a study found that tattoos primarily serve hedonistic purposes, with a minority seeking medical (0.5%) or cosmetic (3%) purposes [4]. Tattooing, involving the introduction of pigments into the dermis, can be decorative, traumatic, cosmetic, or medical, with procedures performed by professionals or beauticians [5]. This includes permanent makeup (PMU) for aesthetic enhancement and medical tattooing for skin restoration [5]. In addition to these two types of tattoos [5], new sensory applications of tattoos have emerged recently, such as sensors [6].

### 1.1. The New Frontiers Offered by Tattoos Used as Sensors

In the ever-evolving realm of tattoos, a truly captivating and revolutionary development has emerged in the form of sensing tattoos, also known as electronic tattoos (e-tattoos) as emphasized by Dhond et al. [6]. These tattoos not only grapple with challenges inherited from the traditional realms of tattooing but also introduce a new dimension by seamlessly integrating with cutting-edge technology. A more thorough exploration of the web, employing the seemingly straightforward key phrase “sensing tattoos”, reveals a groundswell of interest in this avant-garde practice. This interest is vividly demonstrated through numerous online pages dedicated to its exploration and discourse, as illustrated by some, albeit not exhaustive, examples [7,8,9,10,11,12]. The mere existence of these dedicated online resources underscores the growing interest and enthusiasm surrounding the paradigm shift introduced by sensing tattoos. This provides compelling evidence of a notable transition towards the exploration and adoption of these innovative applications, marking a pivotal moment in the ever-evolving narrative of tattoo artistry.

#### 1.1.1. Electronic-Tattoos: Pioneering a Paradigm Shift in the Health Domain

All of this suggests a significant new paradigm according to Dhond et al. [6] in the health domain, based on a dual perspective:

- Rethinking Point-of-Care Testing: Moving away from the historical focus on acute medical issues, there is an imperative to revolutionize our approach to healthcare by embracing proactive, long-term disease management. The emergence of dermal tattoo biosensors, serving as continuous monitoring platforms, not only signifies a leap forward in technology but holds the promise of transforming how we diagnose and treat diseases. A thorough exploration of these biosensors is not just about enhancing medical outcomes; it is a potential game-changer in point-of-care diagnostics. The integration of these innovative technologies not only could improve health outcomes but also has far-reaching economic implications, promising significant cost reductions in the long run.
- Rethinking the Skin as a Diagnostic Platform: The skin, often overlooked as more than a protective barrier, emerges as a fascinating frontier for diagnostics. As the body’s largest organ with complex layers, the skin becomes a canvas for innovation in healthcare. By unlocking the skin’s potential as a diagnostic platform, especially in its role as a host for biosensors, we open doors to a new era of continuous monitoring. This research direction signifies more than just a shift; it is a paradigmatic change in how we view traditional diagnostic approaches. The skin, once seen as a passive organ, now stands as an active and dynamic player in the diagnostic landscape, showcasing its potential to revolutionize healthcare practices and improve patient outcomes.

#### 1.1.2. E-Tattoos: A Promising Frontier for Theragnostic Advancements

Sensing tattoos, sensor tattoos, or e-tattoos (various terms for these devices chosen by authors) are increasingly recognized today as a promising approach to real-time theragnostics according to Williams and Franklins’ study [13]. The authors [13] significantly broaden the concept of a sensor tattoo discussed by Dhong [6], introducing the umbrella term ‘e-tattoo’. This term encompasses more sophisticated sensor solutions, going beyond traditional tattoos created with special inks that act as sensors/actuators. The proposed e-tattoos not only rely on engraved tattoos but can also integrate advanced electronics, wearable devices (e.g., special patches), and innovative materials applied directly to the skin’s surface.

Theragnostics represents an innovative fusion of “therapy” and “diagnostics”, embodying a groundbreaking approach that marries therapeutic and diagnostic capabilities within a singular medical intervention. This cutting-edge concept aims to transcend traditional medical boundaries by seamlessly integrating diagnostic tools with therapeutic agents, offering a holistic and personalized approach to patient care.

In practical terms, theragnostics involves the development and utilization of diagnostic tools with inherent therapeutic properties or the incorporation of therapeutic agents that concurrently provide diagnostic insights. This convergence of diagnostics and therapy holds immense promise, particularly in fields like cancer treatment, where it enables the precise tailoring of interventions based on an individual’s unique characteristics.

The emphasis on theragnostics underscores a paradigm shift in medicine, moving towards highly personalized and targeted treatments. By intertwining diagnostic precision with therapeutic efficacy, this approach not only enhances treatment outcomes but also holds the potential to minimize adverse effects. In essence, theragnostics is a pioneering frontier in healthcare, ushering in a new era of integrated and individualized medical strategies.

Williams and Franklin [13] have carefully emphasized the innovative potential inherent in e-tattoos in the field of theragnostics, based on a comprehensive understanding of critical considerations that we briefly summarize below. Expanding on their insights [13], a significant challenge in modern medicine lies in the sporadic measurement of vital biomarkers, especially within the blood, coupled with the standardized dosing of medications. The simultaneous continuous monitoring of pertinent analytes and precise drug dosing holds the potential to profoundly impact treatment strategies and overall health, heralding a potential revolution in medicine [14,15].

This transformative potential is particularly crucial for managing chronic diseases such as diabetes, where frequent monitoring of blood sugar levels is essential for effective symptom management [16]. Williams and Franklin [13] also suggest that similar capabilities could reshape the landscape for numerous chronic and acute conditions, including Crohn’s disease and complications due to heart failure. Despite recent advancements in wearables, exemplified by smartwatches, facilitating non-invasive monitoring of limited ex vivo patient data, it is evident that the true paradigm shift toward widespread continuous health monitoring is still evolving, as underscored in [13,17,18].

**Figure 1:**
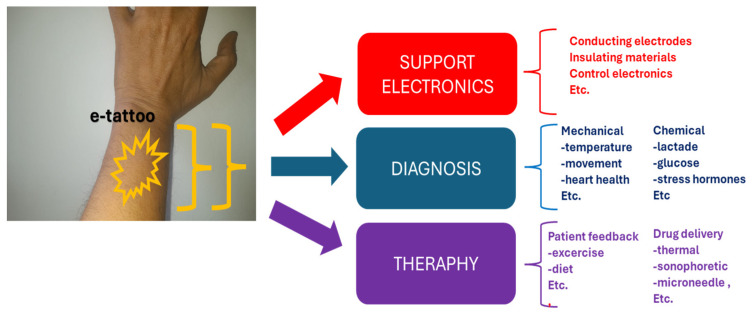
E-tattoo in theragnostics.

Advancements in biocompatible materials and sensors are rapidly expanding the horizons of devices for continuous monitoring and the regulation of chronic symptoms. Various modalities support continuous monitoring and dosing, including wearables, ingestibles, or implantables, and on-skin electronics, colloquially referred to as electronic tattoos [19,20]. While wearables are ubiquitous, Williams and Franklin [13] assert that their limited contact with the skin may hinder the detection of certain biological signals. Ingestible electronic biomonitoring excels in detecting chemical signals within the biological environment but faces challenges in controlling location post-ingestion [21,22]. Implantable electronics present challenges of biofouling due to the foreign body response [13,23]. E-tattoos, as illuminated by Williams and Franklin [13], represent non-permanent devices intimately placed on the skin. This innovative approach, combining comfort and precision, mirrors the aesthetics of temporary tattoos, offering a unique avenue for continuous monitoring and therapy. E-tattoos directly monitor biological signals through the epidermal layer and facilitate therapeutic drug transfer via the dermis, presenting a potential shift from a diagnosis-only model to a comprehensive theragnostic system [13,24]. Their lifespan is typically considered to be a few days at the shortest [25], aligning with the cycle length of desquamation of the outermost layer of the dermis, approximately 20–30 days [26]. To develop e-tattoos for continuous monitoring and therapy, Williams and Franklin [13] propose that three key components (Figure 1) must synergize:

- Biological sensing/diagnostics;
- Drug delivery/therapeutics;
- Robust support system.

While sensing and dosing take center stage, Williams and Franklin [13] emphasize that the support system, encompassing electrodes, processing, encapsulation, and filtering, is equally critical to functionality. Figure 1 illustrates a schematic representation of the application and key elements of an e-tattoo in theragnostics.

### 1.2. The Rationale for a Review Study and the Purpose

#### 1.2.1. The Rationale for a Review

All that has been outlined above highlights how inherited and emerging issues in sensing tattoos present a diverse set of challenges. Inherited problems encompass allergies, infections due to inadequate sterilization, fading over time, complex removal processes, and societal considerations [27,28]. New challenges involve technical complexity, power supply management, long-term effects on skin health, individual sensitivity variations, and privacy concerns. This comprehensive overview emphasizes the necessity for a thorough review. Key emerging questions center around technological advancements, the effectiveness of sensory tattoos, their adoption rate, long-term health implications, individual responses, privacy safeguards, and the establishment of robust standards and regulations in this dynamic field.

#### 1.2.2. Purpose of the Study

The primary objective of this study is to undertake a comprehensive narrative review within the realm of sensing tattoos. Through an exploration of the existing literature and advancements in the field, this review aims to provide a thorough understanding of the current state of sensing tattoos, including their technological underpinnings, applications, and potential implications. By synthesizing and critically analyzing available information, the study seeks to offer valuable insights into the evolving landscape of sensing tattoos, contributing to the collective knowledge and fostering future developments in this innovative intersection of technology and body art.

## 2. Methods

The narrative review used the ANDJ standardized checklist designed for narrative reviews [29]. The Pubmed and Scopus databases were inserted in the overview. A qualification methodology was used to choose the studies, based on the assessment of qualified parameters [30]. Based on [29], we evaluated each contribution based on key parameters:

- N1: Clarity of study rationale in the introduction.
- N2: Appropriateness of work’s design.
- N3: Clarity in describing methods.
- N4: Clear presentation of results.
- N5: Justification and alignment of conclusions with results.
- N6: Adequate disclosure of conflicts of interest by authors.

We assigned a graded score (1 to 5) for N1–N5.

For N6, we provided a binary assessment (Yes/No) for disclosure of conflicts.

Preselect studies meeting criteria:

N6 must be “Yes” for conflict disclosure.

The score for parameters N1–N5 must always be greater than the threshold (Th) 3.

Only peer-reviewed studies were considered (including congress proceedings if peer reviewed).

We conducted thorough research, directing our attention towards both skin and tattoos. This investigation was conducted in tandem with an **AND** Boolean operation, specifically involving sensor, biosensor, sensing, or biosensing.

Our exploration encompassed an analysis of both the “title/abstract” and the “full text” of relevant studies.

**Figure 2:**
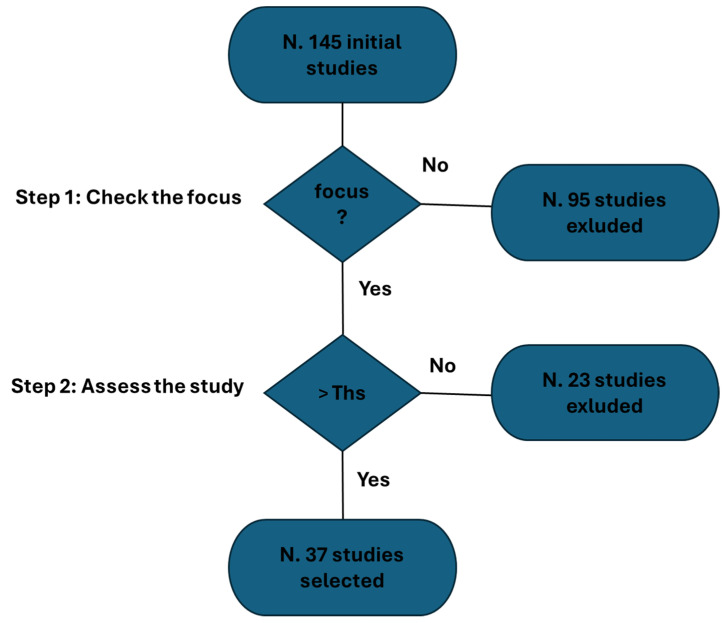
Study selection process.

Furthermore, we aspire to illustrate the process of study selection through a detailed diagram (Figure 2). It is noteworthy to emphasize that, despite the visual aid, our review maintains a purely narrative nature (it is not a systematic review). The process depicted in Figure 2 unfolded in two distinct steps:

- Initial Screening Process: A meticulous screening process was implemented to validate the authentic emphasis on e-tattoos. This step was crucial in ensuring the precision and relevance of the selected studies.
- Application of Selection Algorithm: Subsequently, in the second step, we applied a rigorous selection algorithm based on the parameters mentioned earlier.

At the outset, 145 studies were identified (Figure 2). Of these, 95 studies were excluded due to a lack of focus. An additional 23 studies were excluded following the evaluation process. The procedure-based overview identified 37 studies.

The detected studies are the following ones [31,32,33,34,35,36,37,38,39,40,41,42,43,44,45,46,47,48,49,50,51,52,53,54,55,56,57,58,59,60,61,62,63,64,65,66,67]. Nine of these studies are reviews [31,36,38,39,49,52,56,66,67].

## 3. Results

The results are presented separately for scientific studies and reviews. Below is a comprehensive analysis of the following: (I) The scientific studies, divided into a detailed analysis (Section 3.1) and key findings (Section 3.2). (II) The reviews, also separated into a detailed analysis (Section 3.3) and key findings (Section 3.4).

### 3.1. In-Depth Analysis of the Detected Studies: A Comprehensive Overview

Minwoo et al. [32] introduced affordable and user-friendly disposable wearable sensors, featuring ultrathin conformable films transferred onto human skin using glossy paper (GP) and liquid bandages (LB). The method involves spray-coating silver nanowire (AgNW) composite films onto GP, which easily adhere to the skin due to GP’s hydrophobic and rough surface. The LB ensures stable attachment, enabling continuous recording of electrophysiological signals like an electromyogram (EMG), electrocardiogram (ECG), and electrooculogram (EOG). The LB promotes rapid adhesion and environmental stability, while the AgNW composite exhibits high breathability, making it suitable for extended use in creating affordable tattoo-like sensors.

Wang et al. [33] addressed the unmet need for noninvasive venous blood oxygenation monitoring. They introduced a soft wearable e-tattoo sensor that simultaneously measures arterial and venous pulses from the wrist. The study explores the challenge of crosstalk between arterial and venous signals and proposes spatial filtering as a solution. This novel method aims to enhance clinical diagnoses of conditions like sepsis and shock by providing simultaneous measurements of arterial and venous blood oxygenation.

Lim et al. [34] focused on surface electromyography (sEMG) sensors and introduced an integrated system called an electronic tattoo (E-Tattoo). This wearable, flexible epidermal sensor improves long-term comfort by intimately attaching electrodes to the skin. The prototype demonstrates effectiveness in monitoring muscle activities, providing comparable signal quality to commercial products with enhanced comfort and signal-to-motion artifact ratio during active movements.

Vural et al. [35] conducted a perspective study on the development of wearable devices for comprehensive and accurate health monitoring. They emphasize the importance of noninvasive electrochemical sensors in detecting biomarkers in body fluids, such as tears, saliva, perspiration, and skin interstitial fluid (ISF). The review covers recent articles, examining wearable sensors for various biofluids and discussing implementation challenges and future prospects.

He et al. [37] proposed a colorimetric dermal tattoo biosensor for multiplexed detection of health-related biomarkers in skin interstitial fluid. The biosensor, fabricated with a segmented microneedle patch, exhibits color changes in response to biomarker concentration changes (pH, glucose, uric acid, and temperature). This dermal tattoo biosensor shows potential for simultaneous detection of multiple biomarkers in vitro, ex vivo, and in vivo, offering long-term health monitoring capabilities.

Chen et al. [40] presented electronic tattoos as lightweight and noninvasive wearable electronics. They overcame challenges in flexibility, skin biocompatibility, adhesion, repairability, and erasability by using a dynamic ionic liquid. The electronic tattoo, firmly attached to human skin, demonstrated excellent sensing performance in response to temperature variation and tensile strain, intelligently monitoring body temperature, pulse, and movement.

Zhao et al. [41] developed highly conductive graphene nanosheet film-based tattoo dry electrodes (TDEs) for human electrophysiology and strain sensing. The multilayer graphene nanosheet film provided support for fabricating low-cost, customizable, and high-performance electronic tattoos. TDEs exhibited lower skin-electrode contact impedance, enabling accurate detection of electrocardiogram and electromyogram during 24 h wearing.

Gogurla et al. [42] utilized natural silk protein with carbon nanotubes to create an epidermal electronic tattoo (e-tattoo) system for multifunctional applications. The e-tattoo integrated electrically and optically active heaters, a temperature sensor, a stimulator for drug delivery, and real-time electrophysiological signal detectors onto human skin. This approach offers a next-generation electronic platform for wearable and epidermal applications in healthcare.

Taccola et al. [43] developed ultra-conformable temporary tattoo electrodes (TTEs) for electrophysiological recordings. They utilized ink-jet printing of PEDOT:PSS on temporary tattoo paper, demonstrating real-time monitoring of respiration through transthoracic impedance measurements. The proposed interconnection strategy ensured stability and re-positionability, making TTEs suitable for large-scale production and diverse bioelectric signal monitoring.

Kedambaimole et al. [44] showcased a Ti_3_C_2_-MXene resistor as an ultrathin skin-mountable temporary tattoo for highly sensitive strain sensing. The sensor’s skin conformability allows inconspicuous monitoring of vital health parameters, such as pulse rate, respiration rate, and surface electromyography. Its high sensitivity is attributed to nanocrack development upon strain, offering a fast, repeatable, and easily patternable sensor.

Sempionatto et al. [45] explored wearable chemical sensors for personalized nutrition solutions. They presented an epidermal biosensor for noninvasive electrochemical detection of sweat vitamin C, using ascorbate oxidase on flexible printable tattoo electrodes. The biosensor demonstrated selective response, mechanical resiliency, and potential for personalized nutrition assessments by monitoring vitamin C dynamics in sweat.

Laroscelle et al. [46] explored the use of UV-excited luminescent tattoo inks as cost-effective fiducial markers in radiotherapy patient alignment. The study aimed to demonstrate the feasibility of visualizing these inks under megavoltage (MV) radiation, providing a direct visualization of field position during beam delivery. Nine UV-sensitive tattoo inks with various emission spectra were surveyed, and both liquid solutions and skin-simulating phantoms were imaged under MV excitation. The UV inks exhibited peak fluorescence emission between 440 and 600 nm, with lifetimes around 11–16 μs. Despite a sixfold increase in luminescence intensity during the x-ray pulse, the signal-to-noise ratio improvement was only twofold. The study achieved a spatial resolution of 1.6 mm accuracy in skin test phantoms, and optical filtering allowed continuous imaging with a cobalt source, offering color discrimination. The results suggest the potential use of low-cost inks for real-time field verification during MV dose delivery; although, further studies are needed to explore its application as a tool for radiation dosimetry.

Chen et al. [47] developed a transient epidermal sensor using a water-soluble polyethylene oxide (PEO) substrate and a poly(3,4-ethylenedioxythiophene) polystyrene sulfonate (PEDOT:PSS) conjugated polymer. The sensor exhibited stable electronic properties under static stress, dynamic load, and transient conditions. It maintained electrode resistance of up to 2% strain, gradually increased within 6.5% strain under static stress, and demonstrated a fast response to dynamic loads. The PEO substrate dissolved in water, leaving the PEDOT:PSS electrode intact, forming a soft, Van der Waals force-attached, tattoo-like epidermal sensor.

Kim et al. [48] proposed a quantitative imaging method to monitor tattoo removal using laser treatment. The study employed Sprague Dawley rat models tattooed with various ink concentrations, treated with a 755 nm laser over six weeks. The proposed method accurately quantified tattoo contrast variations post-laser treatment. Histological analysis confirmed significant ink removal without thermal injury, showcasing the potential of this monitoring technique for objective assessment in clinical settings.

Ha et al. [50] introduced a stretchable, ultrathin seismocardiography (SCG) sensing e-tattoo based on a 28-µm-thick piezoelectric polymer, PVDF. The soft SCG sensor, integrated with gold electrodes, formed an electromechano-acoustic cardiovascular (EMAC) sensing tattoo. It allowed synchronous ECG and SCG measurements, correlating systolic time interval (STI) with blood pressure. The EMAC tattoo demonstrated reduced motion artifacts and potential for continuous, noninvasive blood pressure estimation.

Kim et al. [51] presented a wearable epidermal platform for simultaneous, noninvasive sampling and analysis of sweat and interstitial fluid (ISF) using a single device. Utilizing a screen-printing technique with temporary tattoo materials, the platform enabled real-time measurement of biomarkers (sweat-alcohol and ISF-glucose) in human subjects. This dual biofluid sampling and analysis system showcased promising applications in next-generation noninvasive epidermal biosensing.

Park et al. [53] developed a method for cost-effective, large-area fabrication of reduced graphene oxide (rGO) films on flexible polymer substrates. Using a flow-enabled self-assembly approach, GO films were deposited and selectively reduced via laser direct writing, demonstrating programmable circuit printing without photolithography. The resulting rGO-based electrical circuit boards exhibited compatibility with electronic module chips and flexible humidity sensors.

Stier et al. [54] introduced a large-area, ultra-thin, ultra-soft tattoo-like heater with autonomous PID temperature control. The device, fabricated using a “cut-and-paste” method, comprised a stretchable aluminum heater and a stretchable gold resistance temperature detector. It demonstrated the ability to maintain a target temperature, adjust to new set points, and conform to skin deformation without imposing constraints, making it suitable for long-term wearability in medical applications.

Mishra et al. [55] developed flexible epidermal tattoo and textile-based electrochemical biosensors for vapor-phase detection of organophosphorus nerve agents. These wearable sensors, based on stretchable organophosphorus hydrolase (OPH) enzyme electrodes, offered rapid and selective square-wave voltametric detection of OP vapors. The stress-enduring inks used for printing the electrodes ensured resilience against mechanical deformations, providing potential applications in decentralized security for rapid warning of personal exposure to OP nerve-agent vapors.

Ameri et al. [57] presented graphene electronic tattoos (GET) as sub-micrometer thick, multimodal sensors designed for wearability. GET, fabricated through a “wet transfer, dry patterning” method, offered high transparency, stretchability, and breathability. Functioning as dry electrodes, the GET–skin interface impedance matched medically used silver/silver-chloride (Ag/AgCl) gel electrodes but with superior comfort, mobility, and reliability. GET demonstrated successful application in measuring various physiological parameters, including ECG, EMG, EEG, skin temperature, and hydration.

Jeong et al. [58] introduced a low-cost, wireless, stretchable biosensor integrating temperature sensor, light source/sensor, NFC chip, and antenna. Fabricated using a “cut-and-paste” method, the biosensor was imperceptible, adhering to skin without mechanical failure. It demonstrated high-fidelity sensing, making it suitable for applications like skin thermography and photometry.

Gong et al. [59] developed highly sensitive, wearable strain sensors using polyaniline microparticles and gold nanowire (AuNW) films. The sensors exhibited enhanced conductivity and sensitivity, and their stretchability was improved by designing curved tattoos with different radii of curvature. Roller coating encapsulation ensured water resistibility and durability. Due to improved conductivity, these sensors directly interfaced with wireless circuitry, enabling applications in human finger-controlled robotic arm systems.

Bandodkar et al. [60] demonstrated an all-printed temporary tattoo-based glucose sensor for noninvasive glycemic monitoring. Combining reverse iontophoretic extraction of interstitial glucose with an enzyme-based amperometric biosensor, the tattoo sensor exhibited a linear response to glucose levels. In vivo testing on human subjects showed promising results, indicating the potential of this tattoo sensor for efficient diabetes management.

Bandodkar et al. in another study [61] developed an epidermal potentiometric sodium sensor embedded in a temporary-transfer tattoo for real-time monitoring of sodium in human perspiration. The wearable device displayed a rapid response, negligible carryover effects, and resilience against mechanical deformations. On-body testing during exercise demonstrated continuous monitoring of sweat sodium dynamics, highlighting its potential for applications in healthcare, fitness, military, and skincare.

Wang et al. [62] discusses the rising interest in wearable electronics for applications like intelligent sensors and artificial limbs. It introduces a novel solution, a versatile electronic tattoo (e-tattoo), utilizing a unique mixed-dimensional matrix network with MXene nanosheets and cellulose nanofibers/Ag nanowires. This e-tattoo offers exceptional sensing capabilities for temperature, humidity, strain, proximity, and material identification. Its multidimensional design enables easy fabrication on diverse substrates using hybrid inks and various methods. Notably, the e-tattoo’s triboelectric properties make it a potential power source for small electronic devices. The authors envision these adaptable e-tattoo systems as a promising platform for the next generation of wearable and epidermal electronics, addressing challenges in skin conformity and functionality.

Jang et al. [63] introduces a solution for unobtrusive electrodermal activity (EDA) sensing for mental stress using imperceptible graphene e-tattoos (GET) on the palm. Existing EDA sensors face issues of obstructiveness or low signal fidelity. To enable ambulatory use, the authors propose heterogeneous serpentine ribbons (HSPRs), minimizing strain concentration at interfaces. HSPR, overlapping GET with a gold serpentine, demonstrates a significant strain reduction. Combined with a soft interlayer, this allows for effective ambulatory EDA monitoring on the palm in real-life conditions. The study also introduces a novel EDA event selection policy, validating the GET EDA sensor against established gold standard.

Galliani [64] focuses on the integration of wearable electronic devices for monitoring physiological signals, particularly in healthcare applications. It introduces a method for creating conformable thin sensors using cost-effective and scalable processes, specifically cutaneous electrode patterning with PEDOT:PSS on wearable substrates. The emphasis is on achieving high-quality recordings and long-term functionality on the human body. The text includes details on electrode characterization through impedance spectroscopy to evaluate signal transduction performance. The need for comparative studies against clinical gold standards is highlighted. The protocol provided enables biosignal recordings in various configurations using a portable electronic setup, aiming to contribute to the advancement of wearable sensors for human health monitoring.

Tang et al. [65] introduces a groundbreaking electronic tattoo for health and movement sensing on the skin, achieving remarkable characteristics like high stretchability, conformality, and adhesion. Notably, it incorporates the crease amplification effect, tripling the output signal from strain sensors. The tattoo can be effortlessly transferred to various surfaces, ensuring secure attachment without solvents or heat. Fabrication is simplified and scalable, involving a layer-by-layer approach with metal–polymer conductors and elastomeric block copolymers. A practical application includes a three-layered tattoo with a heater and 15 strain sensors, enabling functions like temperature adjustment, movement monitoring, and remote control of robots.

In the Supplementary Material, a summary table of the emerged elements (described previously more extensively) in this part of the overview is provided.

### 3.2. Common Findings and Key Emerging Technological Theragnostic Approach

From these studies, we can identify more general common findings and more specific emerging themes with the focus to the technology.

The evolution of electronic tattoos (e-tattoos) signifies a transformative journey within wearable and epidermal electronics. At its core, this trajectory is characterized by a shared commitment to overcoming challenges and enhancing usability.

- *Affordability and User-Friendly Design*
  Minwoo et al. [32] pioneered the field by introducing disposable wearable sensors featuring ultrathin conformable films. Their approach, emphasizing affordability and user-friendly design, involved applying silver nanowire (AgNW) composite films onto glossy paper (GP) with liquid bandages (LB) for stable attachment, enabling continuous recording of electrophysiological signals.
- *Noninvasive Monitoring*
  A prevalent theme across various studies is the pursuit of noninvasive monitoring. Wang et al. [33], Lim et al. [34], and Vural et al. [15] explore applications such as venous blood oxygenation, surface electromyography (sEMG) for muscle activity monitoring, and comprehensive health monitoring through noninvasive electrochemical sensors. These studies collectively contribute to technologies prioritizing patient comfort and accessibility.
- *Multiplexed Detection and Biosensing*
  He et al. [37] and Zhao et al. [41] significantly contribute to the realm of multiplexed detection of health-related biomarkers. He et al. propose a colorimetric dermal tattoo biosensor, while Zhao et al. develop highly conductive graphene nanosheet film-based tattoo dry electrodes (TDEs) for electrophysiology and strain sensing, enabling simultaneous and precise detection of multiple biomarkers.
- *Skin Conformability and Comfort*
  Chen et al. [40] and Jang et al. [63] address challenges related to flexibility, skin biocompatibility, adhesion, and comfort. Chen et al. achieve skin conformity using a dynamic ionic liquid, resulting in electronic tattoos firmly attached to human skin. Jang et al. introduce imperceptible graphene e-tattoos (GET) for unobtrusive electrodermal activity (EDA) sensing, emphasizing minimal strain concentration for enhanced comfort during ambulatory monitoring.
- *Advanced Materials and Fabrication Techniques*
  The incorporation of advanced materials and fabrication techniques propels e-tattoo development. Gong et al. [59], Wang et al. [62], and Tang et al. [65] exemplify this trend. Gong et al. create highly sensitive, wearable strain sensors using polyaniline microparticles and gold nanowire (AuNW) films. Wang et al. introduce a versatile electronic tattoo (e-tattoo) using MXene nanosheets and cellulose nanofibers/Ag nanowires, while Tang et al. present a groundbreaking electronic tattoo for health and movement sensing with a layer-by-layer approach using metal–polymer conductors and elastomeric block copolymers.
- *Functional Diversity*
  Studies such as Gogurla et al. [42], Bandodkar et al. [60,61], and Sempionatto et al. [45] showcase the diversity of functionalities. Gogurla et al. integrate electrically and optically active components onto human skin, Bandodkar et al. develop tattoo-based sensors for noninvasive glycemic and sodium monitoring, and Sempionatto et al. explore wearable chemical sensors for personalized nutrition solutions.

In this collective exploration, these technological nuances converge to redefine the landscape of electronic tattoos, highlighting a shared vision of enhanced usability and functionality within the realm of wearable electronics.

*It is intriguing to delve into the details based on these studies to understand the emerging approaches in theragnostics from a technological and procedural standpoint in the use of e-tattoos.*

Table 1 presents the key findings, with a particular emphasis on the technology.

It is also possible to extract a categorization based on the type of sensor. Table 2 reports the categorization.

### 3.3. In-Depth Analysis of the Detected Reviews: A Comprehensive Overview

Makinia et al. [31] introduced sensitive and cost-effective skin piezoelectric sensors combined with organic electrochemical transistors (OECTs) for real-time monitoring of electrophysiological signals. The fully screen-printed (SP) piezoelectric sensors, manufactured on tattoo paper substrates, are integrated with all-printed OECTs using various printing techniques. This innovation allows for the transfer of the SP piezoelectric sensor to the skin, enabling radial pulse monitoring. The results pave the way for the development of all-printed, fully conformable wearable devices with high sensitivity.

Manasa et al. [36] focused on diabetes mellitus, their review explores the evolution of *skin glucose monitoring devices*, emphasizing real-time continuous glucose monitoring systems (rt-CGMs). It highlights the advantages and challenges of skin-patchable glucose monitoring sensors, especially microneedle (MN) array sensory and delivery systems. The comprehensive overview covers material design, assembly techniques, and the potential of a “sense and act” feedback loop. The review discusses current status, limitations, challenges and anticipates future developments in clinical applications.

The review proposed by Sharma et al. [38] delved into nanostructured ion-selective membranes (ISMs) for biomedical applications. It explores design principles, applications, and recent advances, emphasizing the miniaturization of ion-selective electrodes for implantable or wearable devices including smartwatches, *tattoos*, sweatbands, and fabric patches. The article critically reviews developments in miniaturization, sensing, and construction of ISMs over the last decade. It addresses opportunities and challenges in clinical applications, providing recommendations for enhancing accuracy and robustness.

Pazos et al. [39] focusing on traditional *tattoo* inks explored their potential for continuous health monitoring using optical biosensors. It discusses the replacement of tattoo pigments with optical biosensors, emphasizing diagnostic capabilities for diseases and skin conditions. The study highlights the need for further research on tattoo ink composition, related complications, and degradation kinetics. The review discusses clinical advantages, challenges for in vivo implantation, and the potential for self-controlling health management.

Yetisen et al. [49] investigated the intersection of *tattooing* and biosensing. This study introduces minimally invasive, injectable dermal biosensors for monitoring pH, glucose, and albumin concentrations in interstitial fluid. The sensors demonstrate multiplexing capabilities in ex vivo skin tissue, holding potential for managing acid-base homeostasis, diabetes, and liver failure in point-of-care settings.

Valentini et al. [52] focused on Cultural Heritage (CH) fields. This article reviews recent advancements in portable sensor technologies. In particular, the study faces an emerging trend in sensor technology involving the creation of *portable tattoo devices* designed for on-the-spot analysis. This development is particularly crucial when dealing with Cultural Heritage and Art Work objects that are immovable and intangible. The new proposed portable contact sensors (directly applied to art work objects and surfaces) are non-invasive and non-destructive to the different materials and surfaces of which cultural heritage is composed. It covers nanomaterials, miniaturized sensors, wireless signal transmission, and introduces a novel trend of movable tattoo sensor devices for the in situ analysis. The proposed portable contact sensors are non-invasive and non-destructive, offering applications directly to art objects and surfaces.

Michard et al. [56] explored the role of smartphones and electronic tablets in surgical care. This review identifies their potential from pre rehabilitation to rehabilitation. Digital applications, including Apps, serious games, and text messaging, offer assistance in controlling preoperative risk factors. Connected sensors enable real-time monitoring, presenting opportunities for early detection of complications in hospital and home settings. Following surgery, smartphones and/or linked sensors (such as pulse oximeters, adhesive patches, *electronic tattoos*, and bioimpedance necklaces) can be employed for the monitoring of body temperature, heart rate, heart rate variability (for detecting cardiac arrhythmia), respiratory rate, arterial oxygen saturation, and thoracic fluid content. Overall, this review explores the use of smartphones and tablets as ultrasound devices during medical procedures and highlights the contribution of electronic checklists as Apps in enhancing communication and tracking each step of the surgical journey. The review calls for further studies to assess the impact of digital tools on surgical outcomes and postoperative complications.

Zhang et al. [66] highlight that the annual global cost of diabetes care exceeds USD 1 trillion, with over USD 327 billion spent in the United States. Despite some advances, diabetes care technology has seen little change in recent decades. The rise of wearable electronics and novel materials presents an opportunity for the next generation of closed-loop diabetes care. Wearable glucose sensors, embedded in platforms like skin or on-tooth tattoos, patches, eyeglasses, contact lenses, fabrics, mouth guards, and pacifiers, offer non-invasive and real-time glucose analysis in ambulatory settings. These sensors can integrate with implantable drug delivery systems, forming self-regulating closed-loop systems for diabetes management. This review explores emerging trends and innovations in wearable glucose monitoring and implantable insulin delivery technologies, emphasizing advanced materials and construction. It also addresses current challenges, motivating future technological development for improved patient care in diabetes management.

Piro et al. [67] extensively explores advancements in wearable skin chemical sensors across five key applications: sweat analysis, skin hydration, skin wounds, perspiration of volatile organic compounds, and general skin conditions. It details the detection of relevant analytes, discussing transduction principles and sensor performance for each application. Special attention is given to biological fluid collection, storage, reusability, and device lifespan. The analysis highlights existing performance gaps and proposes future directions to bridge them, aiming for effective commercialization of these sensors.

In the Supplementary Material, a summary table of the emerged elements (described previously more extensively) in this part of the overview is provided.

### 3.4. Common Findings and Key Emerging Technological Theragnostic Approach in the Reviews

The studies collectively showcase the transformative potential of tattoo-based technologies across various domains. Makinia et al. [31] introduce skin piezoelectric sensors combined with organic electrochemical transistors for real-time electrophysiological signal monitoring. Manasa et al. [36] focus on the evolution of skin glucose monitoring devices, emphasizing microneedle array sensory systems for continuous glucose monitoring. Sharma et al. [38] delve into nanostructured ion-selective membranes for implantable or wearable devices, discussing opportunities and challenges in clinical applications.

Pazos et al. [39] explore the use of traditional tattoo inks for continuous health monitoring through optical biosensors, emphasizing diagnostic capabilities. Yetisen et al. [49] introduce minimally invasive, injectable dermal biosensors for monitoring pH, glucose, and albumin concentrations in interstitial fluid. Valentini et al. [52] highlight portable tattoo devices for on-the-spot analysis in Cultural Heritage fields, addressing the immovability of art objects. Michard et al. [56] explore the role of smartphones and electronic tablets in surgical care, utilizing connected sensors, electronic tattoos, and bioimpedance necklaces for real-time monitoring. Zhang et al. [66] discuss the potential of wearable glucose sensors embedded in various platforms for non-invasive and real-time glucose analysis in ambulatory settings, offering closed-loop systems for diabetes management.

Piro et al. [67] extensively review wearable skin chemical sensors across applications like sweat analysis and wound monitoring, emphasizing transduction principles and sensor performance. These studies collectively underscore the broad applications of tattoo-based technologies, providing innovative solutions for real-time monitoring in healthcare, cultural heritage preservation, and chemical sensing.

Analyzing the specified reviews, key themes/patterns and trends emerge:

- *Health Monitoring Integration*
  Makinia et al. [31] and Zhang et al. [66] showcase a strong trend of integrating tattoo-based sensors into health monitoring systems. Makinia focuses on real-time monitoring of electrophysiological signals, while Zhang explores wearable glucose sensors for diabetes care.
- *Focus on Diabetes Care*
  Manasa et al. [36], Sharma et al. [38], and Zhang et al. [66] contribute to the growing emphasis on diabetes care. Manasa reviews the evolution of skin glucose monitoring devices, Sharma explores nanostructured ion-selective membranes, and Zhang discusses wearable glucose sensors, collectively aiming to improve diabetes management.
- *Biosensing Applications*
  Yetisen et al. [49] and Piro et al. [67] delve into biosensing applications. Yetisen introduces injectable dermal biosensors for monitoring pH, glucose, and albumin concentrations, while Piro explores wearable skin chemical sensors across various applications, indicating a trend toward versatile biosensing capabilities.
- *Cultural Heritage and Portable Sensors*
  Valentini et al. [52] introduce a unique perspective by focusing on Cultural Heritage fields. The study emphasizes portable tattoo devices for on-the-spot analysis, contributing to non-invasive and non-destructive examination of art objects, suggesting a novel application beyond healthcare.
- *Technological Impact on Surgery*
  Michard et al. [56] explore the use of smartphones and electronic tablets in surgical care. This review identifies the potential of digital applications and connected sensors for real-time monitoring in preoperative, intraoperative, and postoperative phases, indicating a shift toward digital tools in surgical settings.

These reviews collectively showcase the versatility of tattoo-based technologies, with a notable focus on health monitoring, diabetes care, biosensing, cultural heritage applications, and technological impact on surgical procedures. The trends suggest a broader integration of tattoo-based sensors into diverse fields for enhanced monitoring and diagnostic capabilities.

Review studies play a pivotal role in shedding light on the degree of stabilization achieved by technological solutions, offering a comprehensive analysis of a significant body of scientific research. Table 1 reported previously captures key revelations from these reviews, placing a spotlight on technological processes and advancements.

## 4. Discussion

The discussion is organized into five sections, each rendered into distinct paragraphs. The opening paragraph establishes the context by exploring the prevailing trends in the dissemination of knowledge within this domain. The subsequent paragraph then unfolds in two facets: (I) an examination of the pivotal findings arising from the study results, with a keen focus on discerning emerging opportunities; and (II) an analysis of the limitations and areas demanding a more extensive investigation, aiming to provide a comprehensive perspective. The third paragraph presents and delves into a synoptic graphic that serves as a comprehensive map outlining the overarching message of the narrative review. Moving on to the fourth paragraph, it succinctly delivers key takeaway messages. The final paragraph, the fifth one, outlines the limitations and briefly suggests new avenues for future research.

### 4.1. Numerical Trends in the Tattoo-Based Sensoring

**Figure 3:**
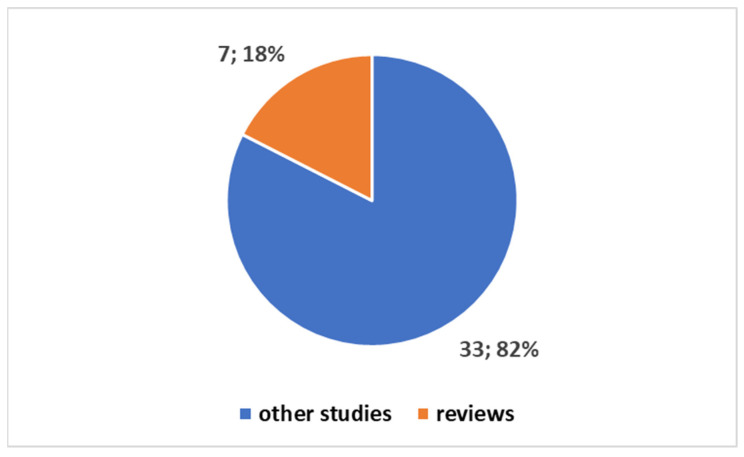
Trends on the studies in the fields on the tattoo-based sensoring.

**Figure 4:**
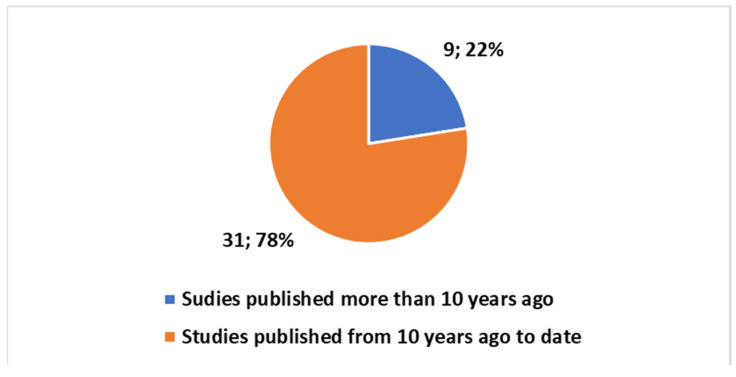
Trends on the studies in the field on the tattoo-based sensoring with reference to the last ten years.

**Figure 5:**
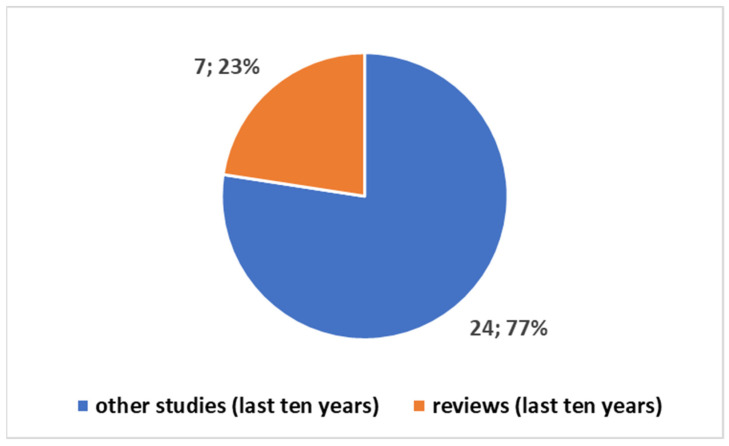
Trends of studies in the last ten years in the tattoo-based sensoring.

Exploring the evolving landscape of scientific publications in this domain, using the first one of the different proposed composite keys, reported in the Box S1 in the Supplementary Material to explore a trend by keyword. We applied the research on the PubMed platform. Figure 3 reports the trends. Notably, only 17% of these publications are reviews. No one of these reviews is a systematic review. Since the presence of systematic reviews in a specific field is an indicator of the consolidation of medical knowledge, the absence of this type of study suggests that this field is still in a more embryonic stage and is open to significant scientific developments. If we focus on the last decade (Figure 4), we observe that 78% of the studies have been produced during this period. All reviews have been generated in the last decade as well (Figure 5).

A further investigation, using the second composite key in Box S1 in the Supplementary Material aimed at identifying any correlation of this trend with the COVID-19 pandemic yields zero results, highlighting a lack of correlation.

### 4.2. Interpretation of Results: Opportunities, Limitations, and Suggestions for a Broader Investigation

The overview presents a dual perspective, one focusing on scientific studies’ intricate details and findings, while the other lens looks at reviews, offering a panoramic view that consolidates and contextualizes collective wisdom. The added value of this approach has been to highlight collective emerging themes globally, along with the opportunities and areas where further investigation is encouraged. In fact, to date, other reviews have addressed specific themes and specific technological solutions among a wider array. A global perspective has allowed for a 360-degree extraction of both emerging approaches in theragnostics and suggestions for specific future research directions.

#### 4.2.1. Opportunities

The exploration of sensing tattoos has revealed a promising landscape with diverse opportunities spanning various thematic areas. One notable avenue is the pursuit of affordability, exemplified by Makinia et al. [31], who introduced sensitive and cost-effective skin piezoelectric sensors. This not only addresses the economic aspect but also underscores the potential democratization of healthcare technologies.

User-friendly design has emerged as a focal point, emphasizing the need for wearables that are not only affordable but also accessible to users. In this context, Minwoo et al. [32] proposed disposable wearables that are both affordable and user-friendly. Similarly, Chen et al. [40] contributed to this theme by presenting electronic tattoos as lightweight, noninvasive wearable electronics.

The exploration of diverse sensorization solutions has been a key trend, with various studies delving into different sensor technologies such as piezoelectric sensors [31], chemical sensors [67], and biosensors [49]. This diversity opens up opportunities for tailoring sensing tattoos to specific applications and improving overall functionality.

A critical aspect of sensing tattoos is the focus on materials. Sharma et al. [38] delved into nanostructured ion-selective membranes (ISMs), addressing challenges related to the longevity and stability of materials used in sensing tattoos. Additionally, Valentini et al. [52] focused on stable and non-destructive portable contact sensors, contributing to enhanced biocompatibility.

Comfortable design has been highlighted in the research, with Laroscelle et al. [46] exploring the use of UV-excited luminescent inks for cost-effective fiducial markers, emphasizing potential comfort during radiotherapy. Chen et al. [47] added to this theme by developing a transient epidermal sensor with a soft, tattoo-like design, addressing concerns related to adhesion and potential discomfort during prolonged wear.

Seamless integration of sensing tattoos with the human body and technology has been a consistent pursuit. Wang et al. [33] introduced a soft e-tattoo for simultaneous pulse measurement, aiming for seamless integration. Likewise, Michard et al. [56] explored the use of smartphones and linked sensors for real-time monitoring, aligning with the broader theme of integration.

The focus on non-invasive health monitoring has been a standout opportunity, with various studies, including Ha et al. [50] and Zhang et al. [66], discussing the application of electronic tattoos for continuous, noninvasive health monitoring. This signifies a shift towards proactive healthcare practices that prioritize continuous monitoring without invasion.

Advancements in imaging and multiplexed detection capabilities have been significant. Laroscelle et al. [46] explored luminescent tattoo inks for imaging in radiotherapy, while Piro et al. [67] discussed multiplexed detection with wearable chemical sensors. These opportunities open avenues for diverse applications in healthcare and related fields.

Table 3 reports among other information the emerging opportunities along with their corresponding studies.

#### 4.2.2. Limitations and Suggestions for a Broader Investigation

The exploration of sensing tattoos, while brimming with opportunities, also encounters several limitations that warrant consideration for a broader investigation. Understanding these constraints is crucial for the balanced development and deployment of this technology.

In the realm of affordability, a primary limitation lies in the potential constraints imposed by the choice of materials and fabrication processes [31]. The delicate balance between cost-effectiveness and the integration of advanced technologies may present challenges, as highlighted by Bandodkar et al. [60]. This limitation underscores the need for meticulous cost management to ensure widespread accessibility.

User-friendly design, although an opportunity, faces challenges in achieving optimal user comfort and wearability. Issues related to adhesion and durability may affect the overall user experience [40,47]. Despite efforts to create lightweight and noninvasive wearables, the trade-off between design and prolonged wear comfort remains an ongoing limitation.

The exploration of diverse sensorization solutions brings about challenges related to variations in sensor performance and reliability across different sensing technologies [67]. Achieving a balance between innovation and consistent sensor performance presents a limitation that necessitates careful consideration in the development process.

Material-focused opportunities, such as the use of nanostructured ion-selective membranes (ISMs) [38] and portable contact sensors [52], come with their own set of challenges. Longevity and stability issues of materials used in sensing tattoos may impose limitations, especially when considering the dynamic and diverse conditions they encounter on the human body.

Comfortable design, although an opportunity, faces potential issues such as adhesion problems and discomfort during prolonged wear [47]. The challenge lies in addressing these issues effectively to ensure that the intended benefits of comfortable, long-term wear are achieved without compromising functionality.

Seamless integration, a critical aspect of sensing tattoos, encounters challenges in achieving integration with smartphones [56]. The potential limitations in real-time monitoring during various surgical stages highlight the need for overcoming integration hurdles to fully harness the capabilities of this technology.

Non-invasive health monitoring, while promising, faces challenges in achieving continuous, real-time monitoring of various health parameters [66]. The potential limitations in the accuracy of blood pressure estimation [50] pose challenges that need to be addressed for widespread adoption in healthcare applications.

Advancements in imaging and multiplexed detection capabilities also bring about challenges. Achieving high-resolution imaging with luminescent tattoo inks, as explored by Laroscelle et al. [46], may face technical constraints. Additionally, potential limitations in the sensitivity of multiplexed detection, as discussed by Piro et al. [67], underscore the need for ongoing research and innovation.

Wearable chemical sensors, despite their potential, face challenges related to the selective detection of specific biomarkers in sweat [45]. Issues regarding the durability and stability of wearable chemical sensors, as highlighted by Gong et al. [59], pose limitations that require careful consideration.

Cost-effective fabrication methods, as demonstrated by Wang et al. [62] and Bandodkar et al. [61], encounter challenges related to scalability for large-scale production. Potential limitations in achieving high-performance sodium sensors [61] highlight the need for refining fabrication processes for broader applicability.

Security applications, as explored by Laroscelle et al. [46] and Piro et al. [67], face challenges in achieving a significant signal-to-noise ratio improvement for security applications. Potential limitations in using luminescent tattoo inks for real-time field verification during MV dose delivery necessitate further exploration.

In summary (see Table 3 with the detected thematic areas), the limitations/suggestions for a broader investigation associated with sensing tattoos encompass aspects of affordability, user-friendly design, diverse sensorization solutions, materials focus, comfortable design, seamless integration, non-invasive health monitoring, imaging, multiplexed detection, wearable chemical sensors, cost-effective fabrication methods, and security applications (Table 3). Identifying and addressing these limitations are integral to realizing the full potential of sensing tattoos in diverse applications.

An overarching consideration on a global scale is the meticulous adherence of e-tattoos, when utilized in theragnostics for routine health applications, to critical facets of medical device regulation, for example, the European medical Device Regulation if used and commercialized in EU [68]. The compliance imperative encompasses factors such as the intended application/destination use, the level of implantability, the power source presence/absence, the specific chemical processes, the material selection, and the nature of data connections, necessitating strict alignment with applicable standards.

For instance, when envisaging e-tattoos for implantable purposes, stringent adherence to medical device regulations becomes paramount to ensure both safety and efficacy according to the destination of use as an implantable device. The regulatory terrain, influenced by factors like implantation duration, invasiveness, and potential health impact, underscores the importance of adhering to established norms for securing regulatory approval and ensuring ethical deployment in clinical settings.

The role of the power supply absent/present source is equally crucial, with external or integrated power prompting the need for compliance with relevant specific regulations.

The deployment of specific chemical processes and materials in e-tattoos requires meticulous attention to regulatory mandates, particularly as these devices come into direct contact with skin or bodily fluids. Adherence to regulations ensures biocompatibility and user safety, emphasizing the necessity for rigorous testing and validation procedures.

Furthermore, the type of data connection used by e-tattoos demands compliance with data protection and privacy regulations. For instance, if the device wirelessly transmits health-related data, it must adhere to established standards to safeguard patient information and maintain confidentiality—compliance with these regulations, a legal requisite, is also a fundamental ethical consideration in the healthcare domain.

The effective integration of e-tattoos into routine healthcare hinges on a profound understanding and adherence to global regulations governing medical devices. This spans considerations encompassing implantability, power supply, chemical processes, material safety, and data connections. Strict adherence to these regulatory frameworks ensures the ethical and responsible deployment of e-tattoos in diverse healthcare settings.

However, it is challenging to disregard the significant themes still unexplored in the genuine integration of these technologies into the health domain.

Many initial questions posed in the justification for this review study remain unanswered, aligning with broader trends indicating the nascency of these technologies.

Scholars are currently more focused on pushing the boundaries of innovation with wearable sensing tattoos, neglecting essential considerations such as medical device standards, regulatory frameworks, and cybersecurity [69,70,71]. In contrast, established implantable devices like pacemakers and cutting-edge innovations like artificial pancreases receive dedicated attention on these critical themes [72,73,74]. This discrepancy underscores a gap in the current trajectory of research and development within the domain of sensing tattoos.

### 4.3. Synoptic Diagram of the Study

**Figure 6:**
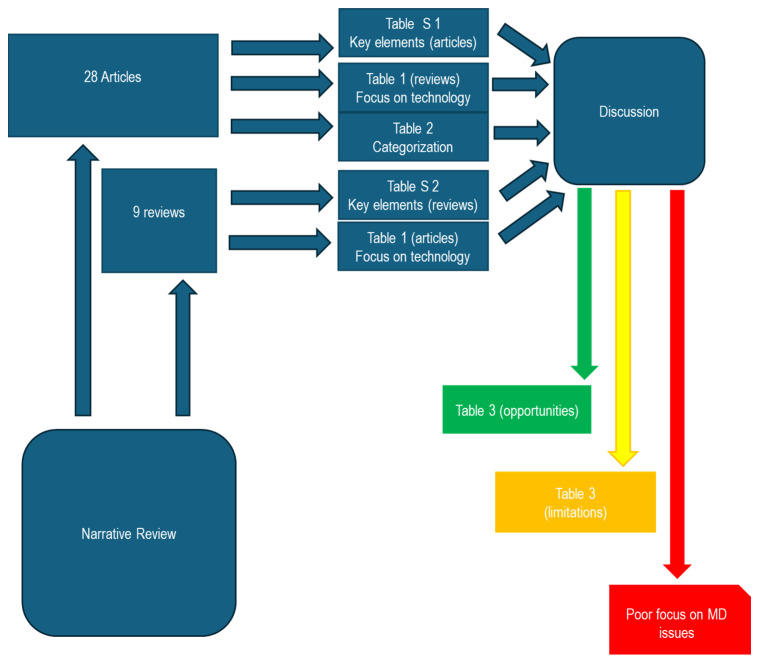
Synoptic diagram.

The diagram presented below (Figure 6) intricately outlines the pivotal connections established within the scope of this study through comprehensive summary tables. The study meticulously analyzed a corpus of 28 scientific articles and 9 review studies, each offering distinct perspectives. Tables S1 and S2 succinctly encapsulate key elements from the analysis of articles and reviews. Table 1 focuses on technological aspects, while Table 2 reports a categorization based on the sensor type. A methodical discourse pathway unfolded, unveiling a spectrum of opportunities (Table 3, opportunities section), shedding light on aspects potentially requiring in-depth analysis (Table 3, limitations section), and highlighting the evident lack of focus on studies delving into aspects closely related to Medical Devices (MDs).

### 4.4. Final Takeaway Message

Sensing tattoos or e-tattoos offer transformative possibilities in healthcare and monitoring, yet their integration faces multifaceted challenges. Key findings highlight promising applications, but critical limitations encompass medical device standards, cybersecurity concerns, safety considerations, regulatory compliance, ethical data usage, user education, interoperability standards, and environmental sustainability. Recommendations for improvement include fostering collaborative ecosystems, prioritizing user-centric design, developing ethical frameworks, advocating for streamlined regulations, implementing education initiatives, and encouraging interdisciplinary research. Successfully navigating these challenges requires a holistic and collaborative approach, ensuring innovative wearables align with user expectations, ethical considerations, and global standards.

### 4.5. Limitations and Future Work

The narrative review conducted a comprehensive analysis by scrutinizing two prominent databases, Pubmed and Scopus. This deliberate choice in database selection was made with the specific aim of honing in on a thorough examination of both the technological developments and their direct applicability to the medical domain. In doing so, the study aimed to provide a nuanced and detailed insight into the current state of the art at the intersection of technology and medicine. The narrative review extracts pivotal developmental themes and illuminates areas in need of further exploration. These insights not only contribute significantly to shaping future research directions but also, in tandem with the ongoing advancement of medical knowledge, lay a robust foundation for the undertaking of targeted studies, including comprehensive systematic reviews.

## 5. Conclusions

In conclusion, the landscape of sensing tattoos presents a myriad of opportunities and challenges that warrant careful consideration for their successful integration into diverse domains. The emphasis on affordability, user-friendly design, and versatile sensorization solutions underscores the potential for these technologies to play a transformative role in health monitoring, environmental sensing, and beyond. However, certain limitations, including issues related to adhesion, signal quality, biocompatibility, and regulatory complexities, highlight the need for continuous refinement. The identified opportunities, such as non-invasive health monitoring, multiplexed detection, and cost-effective fabrication methods, pave the way for innovative applications in personalized healthcare and beyond. Nevertheless, it is crucial to address the existing gaps, particularly in areas of medical device standards, cybersecurity, and regulatory compliance, to ensure the seamless integration and widespread adoption of these technologies. The call for a holistic approach, user-centric design, and interdisciplinary collaboration emerges as a central theme in both opportunities and suggestions for improvement. Bridging the gap between innovation and practicality, prioritizing ethical considerations, and fostering a collaborative ecosystem are essential for the evolution of sensing tattoos. As we navigate the complexities of these technologies, there is a clear indication that the field is in a dynamic state of development, with researchers actively pushing boundaries and exploring new frontiers.

## Figures and Tables

**Table 1:** Technology/technological process in the overviewed studies.

Technology/ Technological Process Faced in the Study	Description	Type of Study, Associated Study
*Ultrathin conformable films transferred onto human skin using glossy paper (GP) and liquid bandages (LBs). Spray-coating of silver nanowire (AgNW) composite films onto GP*	Enables continuous recording of electrophysiological signals (EMG, ECG, EOG). LB ensures stable attachment, promoting rapid adhesion and environmental stability. AgNW composite offers high breathability, suitable for extended use in creating affordable tattoo-like sensors.	Article, [32]
*Soft wearable e-tattoo sensor*	Simultaneously measures arterial and venous pulses from the wrist. Addresses crosstalk between arterial and venous signals by proposing spatial filtering. Aims to enhance clinical diagnoses of conditions like sepsis and shock by providing simultaneous measurements of arterial and venous blood oxygenation.	Article, [33]
*Flexible Electronic Tattoo*	Wearable, flexible epidermal sensor intimately attaches electrodes to the skin, improving long-term comfort. Demonstrates effectiveness in monitoring muscle activities, providing comparable signal quality to commercial products with enhanced comfort and signal-to-motion artifact ratio during active movements.	Article, [34]
*Non-invasive electrochemical sensors*	Emphasizes the importance of electrochemical sensors in detecting biomarkers in body fluids (tears, saliva, perspiration, and skin interstitial fluid). Reviews recent articles, examining wearable sensors for various biofluids, and discusses implementation challenges and future prospects.	Article, [35]
*Segmented microneedle patch*	Multiplexed detection of health-related biomarkers in skin interstitial fluid. Exhibits color changes in response to biomarker concentration changes (pH, glucose, uric acid, and temperature). Shows potential for simultaneous detection of multiple biomarkers in vitro, ex vivo, and in vivo, offering long-term health monitoring capabilities.	Article, [37]
*Dynamic ionic liquid*	Lightweight and noninvasive wearable electronics firmly attached to human skin. Demonstrates excellent sensing performance in response to temperature variation and tensile strain. Intelligently monitors body temperature, pulse, and movement.	Article, [40]
*Highly conductive graphene nanosheet film*	Developed for human electrophysiology and strain sensing. Provides support for fabricating low-cost, customizable, and high-performance electronic tattoos. Exhibits lower skin–electrode contact impedance, enabling accurate detection of electrocardiogram and electromyogram during 24 h wearing.	Article, [41]
*Natural silk protein with carbon nanotubes*	Multifunctional electronic tattoo integrating electrically and optically active heaters, a temperature sensor, a stimulator for drug delivery, and real-time electrophysiological signal detectors onto human skin. Offers a next-generation electronic platform for wearable and epidermal applications in healthcare.	Article, [42]
*Ink-jet printing of PEDOT:PSS on temporary tattoo paper*	Developed for electrophysiological recordings, enabling real-time monitoring of respiration through transthoracic impedance measurements. Proposed interconnection strategy ensures stability and re-positionability, making them suitable for large-scale production and diverse bio-electric signal monitoring.	Article, [43]
*Ti_3_C_2_-MXene resistor*	Ultrathin skin-mountable temporary tattoo for highly sensitive strain sensing. Skin conformability allows inconspicuous monitoring of vital health parameters such as pulse rate, respiration rate, and surface electromyography. High sensitivity attributed to nanocrack development upon strain, offering a fast, repeatable, and easily patternable sensor.	Article, [44]
*Epidermal biosensor with ascorbate oxidase on flexible printable tattoo electrodes*	Explores wearable chemical sensors for personalized nutrition solutions. Enables noninvasive electrochemical detection of sweat vitamin C, showcasing selective response, mechanical resiliency, and potential for personalized nutrition assessments by monitoring vitamin C dynamics in sweat.	Article, [45]
*UV-excited luminescent tattoo inks*	Cost-effective fiducial markers in radiotherapy patient alignment. Demonstrates the feasibility of visualizing these inks under megavoltage (MV) radiation, providing direct visualization of field position during beam delivery. UV-sensitive tattoo inks exhibit peak fluorescence emission between 440 and 600 nm, with lifetimes around 11–16 μs. Despite some challenges, such as a six-fold increase in luminescence intensity during the x-ray pulse, the study achieved a spatial resolution of 1.6 mm accuracy in skin test phantoms, offering potential real-time field verification during MV dose delivery.	Article, [46]
*Water-soluble polyethylene oxide (PEO) substrate and poly (3,4-ethylenedioxythiophene) polystyrene sulfonate (PEDOT:PSS) conjugated polymer*	Development of a transient epidermal sensor with stable electronic properties under static stress, dynamic load, and transient conditions. Maintains electrode resistance up to 2% strain, gradually increases within 6.5% strain under static stress, and demonstrates a fast response to dynamic loads. The PEO substrate dissolves in water, leaving the PEDOT:PSS electrode intact, forming a soft, Van der Waals force-attached, tattoo-like epidermal sensor.	Article, [47]
*755 nm laser treatment on Sprague Dawley rat models tattooed with various ink concentrations*	Proposes a quantitative imaging method to monitor tattoo removal using laser treatment. Accurately quantifies tattoo contrast variations post-laser treatment. Histological analysis confirms significant ink removal without thermal injury, showcasing the potential of this monitoring technique for objective assessment in clinical settings.	Article, [48]
*28 µm thick piezoelectric polymer (PVDF)*	Introduction of a stretchable, ultrathin seismocardiography (SCG) sensing e-tattoo. Integrated with gold electrodes, forming an electro-mechano-acoustic cardiovascular (EMAC) sensing tattoo. Allows synchronous ECG and SCG measurements, correlating systolic time interval (STI) with blood pressure. Demonstrates reduced motion artifacts and potential for continuous, noninvasive blood pressure estimation.	Article, [50]
*Screen-printing technique with temporary tattoo materials*	Presentation of a wearable epidermal platform for simultaneous, noninvasive sampling and analysis of sweat and interstitial fluid (ISF) using a single device. Enables real-time measurement of biomarkers (sweat-alcohol and ISF-glucose) in human subjects. Showcases promising applications in next-generation noninvasive epidermal biosensing.	Article, [51]
*Flow-enabled self-assembly approach*	Development of a method for cost-effective, large-area fabrication of reduced graphene oxide (rGO) films on flexible polymer substrates. Utilizes a flow-enabled self-assembly approach with laser direct writing. The resulting rGO-based electrical circuit boards exhibit compatibility with electronic module chips and flexible humidity sensors.	Article, [53]
*“Cut-and-paste” method with a stretchable aluminum heater and a stretchable gold resistance temperature detector*	Introduction of a large-area, ultra-thin, ultra-soft tattoo-like heater with autonomous PID temperature control. Demonstrates the ability to maintain a target temperature, adjust to new set points, and conform to skin deformation without imposing constraints. Suitable for long-term wearability in medical applications.	Article, [54]
*Stretchable organophosphorus hydrolase (OPH) enzyme electrodes*	Development of flexible epidermal tattoo and textile-based electrochemical biosensors for vapor-phase detection of organophosphorus nerve agents. Offers rapid and selective square-wave voltametric detection of OP vapors. Stress-enduring inks used for printing the electrodes ensure resilience against mechanical deformations, providing potential applications in decentralized security for rapid warning of personal exposure to OP nerve-agent vapors.	Article, [55]
*Sub-micrometer thick graphene electronic tattoos (GET) fabricated through a “wet transfer, dry patterning” method*	GET functions as dry electrodes with high transparency, stretchability, and breathability. Matches medically used silver/silver-chloride (Ag/AgCl) gel electrodes but offers superior comfort, mobility, and reliability. Demonstrates successful application in measuring various physiological parameters, including ECG, EMG, EEG, skin temperature, and hydration.	Article, [57]
*“Cut-and-paste” method*	Introduction of a low-cost, wireless, stretchable biosensor integrating temperature sensor, light source/sensor, NFC chip, and antenna. Imperceptible, adheres to skin without mechanical failure. Demonstrates high-fidelity sensing, suitable for applications like skin thermography and photometry.	Article, [58]
*Polyaniline microparticles and gold nanowire (AuNW) films*	Development of highly sensitive, wearable strain sensors with enhanced conductivity and sensitivity. Stretchability improved by designing curved tattoos with different radii of curvature. Roller coating encapsulation ensures water resistibility and durability. Directly interfaces with wireless circuitry, enabling applications in human finger-controlled robotic arm systems.	Article, [59]
*Reverse iontophoretic extraction of interstitial glucose combined with an enzyme-based amperometric biosensor*	Demonstration of an all-printed temporary tattoo-based glucose sensor for noninvasive glycemic monitoring. Exhibits a linear response to glucose levels. In vivo testing on human subjects shows promising results, indicating the potential of this tattoo sensor for efficient diabetes management.	Article, [60]
*Potentiometric tattoo sensor*	Epidermal Potentiometric Sodium Sensor embedded in a temporary-transfer tattoo on the skin.	Article, [61]
*Mixed-dimensional matrix network with MXene nanosheets and cellulose nanofibers/Ag nanowires*	Introduction of a versatile electronic tattoo (e-tattoo) that utilizes a unique mixed-dimensional matrix network. It offers exceptional sensing capabilities for temperature, humidity, strain, proximity, and material identification. Multidimensional design enables easy fabrication on diverse substrates using hybrid inks and various methods. Triboelectric properties make it a potential power source for a small electronic device.	Article, [62]
*Imperceptible graphene e-tattoos (GET) on the palm with heterogeneous serpentine ribbons (HSPR)*	Solution for unobtrusive electrodermal activity (EDA) sensing for mental stress. HSPR minimizes strain concentration at interfaces, allowing effective ambulatory EDA monitoring on the palm in real-life conditions. Novel EDA event selection policy introduced and validated against established gold standards.	Article, [63]
*Conformable thin sensors created using cutaneous electrode patterning with PEDOT:PSS on wearable substrates*	Focuses on the integration of wearable electronic devices for monitoring physiological signals, particularly in healthcare applications. Introduces a method for creating conformable thin sensors using cost-effective and scalable processes. Emphasis on achieving high-quality recordings and long-term functionality on the human body. The protocol provided enables biosignal recordings in various configurations using a portable electronic setup.	Article, [64]
*Metal–polymer conductors and elastomeric block copolymers in a layer-by-layer approach*	Introduction of a groundbreaking electronic tattoo for health and movement sensing on the skin. Achieves remarkable characteristics like high stretchability, conformality, and adhesion. Incorporates the crease amplification effect, tripling the output signal from strain sensors. Effortlessly transferred to various surfaces, ensuring secure attachment without solvents or heat. Practical application includes a three-layered tattoo with a heater and 15 strain sensors, enabling functions like temperature adjustment, movement monitoring, and remote control of robots.	Article, [65]
*Piezoelectric Sensors with Organic Electrochemical Transistors (OECTs)*	The review introduced sensitive and cost-effective skin piezoelectric sensors integrated with organic electrochemical transistors (OECTs) for real-time monitoring of electrophysiological signals. The fully screen-printed piezoelectric sensors are manufactured on tattoo paper substrates, enabling radial pulse monitoring.	Review, [31]
*Skin Glucose Monitoring Devices*	The study focused on diabetes mellitus, exploring the evolution of skin glucose monitoring devices. Emphasis is placed on real-time continuous glucose monitoring systems (rt-CGMs), particularly microneedle (MN) array sensory and delivery systems.	Review, [36]
*Nanostructured Ion-Selective Membranes*	The overview delved into nanostructured ion-selective membranes (ISMs) for biomedical applications, emphasizing miniaturization for implantable or wearable devices like smartwatches, tattoos, sweatbands, and fabric patches.	Review, [38]
*Optical Biosensors with Traditional Tattoo Inks*	The study explored the potential of traditional tattoo inks for continuous health monitoring using optical biosensors. The study discusses replacing tattoo pigments with optical biosensors for diagnostic capabilities.	Review, [39]
*Injectable Dermal Biosensors for pH, Glucose, and Albumin Monitoring*	The overview investigated minimally invasive, injectable dermal biosensors for monitoring pH, glucose, and albumin concentrations in interstitial fluid, showcasing multiplexing capabilities for managing various health aspects.	Review, [49]
*Portable Sensor Technologies in Cultural Heritage (CH) Fields*	The study focused on Cultural Heritage fields, reviewing advancements in portable sensor technologies. The study introduces portable tattoo devices designed for on-the-spot analysis, especially relevant for immovable and intangible art objects.	Review, [52]
*Smartphones and Electronic Tablets in Surgical Care*	The overview explored the role of smartphones and electronic tablets in surgical care. The review highlights the potential of digital applications and connected sensors for real-time monitoring in various phases of surgical care.	Review, [56]
*Wearable Glucose Sensors and Closed-Loop Diabetes Care*	The study emphasized the global cost of diabetes care and presented wearable glucose sensors embedded in various platforms like skin or on-tooth tattoos, patches, eyeglasses, contact lenses, fabrics, mouth guards, and pacifiers for noninvasive and real-time glucose analysis.	Review, [66]
*Advancements in Wearable Skin Chemical Sensors*	The study extensively explored advancements in wearable skin chemical sensors across applications such as sweat analysis, skin hydration, skin wounds, perspiration of volatile organic compounds, and general skin conditions.	Review, [67]

**Table 2:** Categorization emerged in the studies.

Category	Description	Associated Study
*Health Monitoring and Diagnostics*	Disposable Wearable Sensors for Electrophysiological Signal Monitoring [32] Noninvasive Venous Blood Oxygenation Monitoring [33] Colorimetric Dermal Tattoo Biosensor for Biomarker Detection [37] Wearable Devices for Comprehensive Health Monitoring [35] Electronic Tattoos for Noninvasive Wearable Electronics [40]	[31,32,33,35,37,40]
*Multipurpose and Multifunctional Devices*	Epidermal Electronic Tattoo (E-Tattoo) System [42] Ultra-Conformable Temporary Tattoo Electrodes (TTEs) [43] Ti_3_C_2_-MXene Resistor for Highly Sensitive Strain Sensing [44] Wearable Chemical Sensors for Personalized Nutrition [45]	[42,43,44,45]
*Sensing and Monitoring Techniques*	Quantitative Imaging Method for Tattoo Removal Monitoring [48] Stretchable Seismocardiography (SCG) Sensing E-Tattoo [50] Simultaneous Sampling and Analysis of Sweat and Interstitial Fluid (ISF) [51] Cost-Effective Fabrication of Reduced Graphene Oxide (rGO) Films [53] Large-Area, Ultra-Thin, Ultra-Soft Tattoo-Like Heater [54] Flexible Epidermal Tattoo for Vapor-Phase Detection of Nerve Agents [55]	[48,50,51,53,54,55]
*Wearable Electronics and Biosensors*	Graphene Electronic Tattoos (GET) for Physiological Parameter Monitoring [57] Low-Cost, Wireless, Stretchable Biosensor [58] Wearable Strain Sensors with Polyaniline Microparticles and AuNW Films [59] All-Printed Temporary Tattoo-Based Glucose Sensor [60] Epidermal Potentiometric Sodium Sensor in a Temporary-Transfer Tattoo [61] Versatile Electronic Tattoo (E-Tattoo) with MXene Nanosheets [62] Unobtrusive Electrodermal Activity (EDA) Sensing with Graphene E-Tattoos [63]	[57,58,59,60,61,62,63]
*Miscellaneous*	Large-Area Fabrication of rGO Films with Laser Direct Writing [53] Monitoring Physiological Signals with Conformable Thin Sensors [64] Electronic Tattoo for Health and Movement Sensing with Crease Amplification [65]	[53,64,65]

**Table 3:** Opportunities (in green) and areas needing a broader investigation, i.e., limitations (in red) of the e-tattoos arranged based on the thematic areas.

Thematic Areas	Opportunity/ Limitation	Description	References
Affordability	**Opportunity**	- Makinia et al. [31] introduced sensitive and cost-effective skin piezoelectric sensors. - Bandodkar et al. [60] demonstrated an all-printed temporary tattoo-based glucose sensor for noninvasive glycemic monitoring.	[31,32,33,41,60]
Affordability	**Limitation**	- The cost-effectiveness of sensing tattoos may be limited by the materials used and fabrication processes [31]. - The integration of advanced technologies can potentially increase production costs [60].	[31,33,41,60]
User-Friendly Design	**Opportunity**	- Minwoo et al. [32] introduced affordable and user-friendly disposable wearable sensors. - Chen et al. [40] presented electronic tattoos as lightweight and noninvasive wearable electronics.	[31,32,40,60]
User-Friendly Design	**Limitation**	- Challenges in achieving optimal user comfort and wearability [47]. - Issues related to adhesion and durability may affect user experience [40].	[31,32,40,47,60]
Diverse Sensorization Solutions	**Opportunity**	- Various studies explored different sensor technologies, such as piezoelectric sensors [31], chemical sensors [67], and biosensors [49].	[31,33,41,49,67]
Diverse Sensorization Solutions	**Limitation**	- Variation in sensor performance and reliability across different sensing technologies [67]. - Potential limitations in sensor sensitivity and specificity [49].	[31,33,41,49,67]
Materials Focus	**Opportunity**	- Sharma et al. [38] delved into nanostructured ion-selective membranes (ISMs) for biomedical applications. - Valentini et al. [52] focused on portable sensor technologies, introducing non-invasive and non-destructive portable contact sensors.	[31,38,49,52,67]
Materials Focus	**Limitation**	- Challenges related to the longevity and stability of materials used in sensing tattoos [38]. - Potential concerns regarding the biocompatibility of materials [52].	[31,38,49,52,67]
Comfortable Design	**Opportunity**	- Laroscelle et al. [46] explored the use of UV-excited luminescent tattoo inks for cost-effective fiducial markers, emphasizing potential comfort during radiotherapy. - Chen et al. [47] developed a transient epidermal sensor with a soft, tattoo-like design.	[38,46,47,52]
Comfortable Design	**Limitation**	- Adhesion issues and potential discomfort during prolonged wear [47]. - Suggestion of further focus on Skin irritation or allergic reactions due to materials used [52].	[38,46,47,52]
Seamless Integration	**Opportunity**	- Wang et al. [33] introduced a soft wearable e-tattoo sensor for simultaneous measurement of arterial and venous pulses from the wrist, emphasizing seamless integration. - Michard et al. [56] explored the use of smartphones and linked sensors for real-time monitoring.	[33,38,52,56]
Seamless Integration	**Limitation**	- Integration challenges, especially in achieving seamless integration with smartphones [56]. - Suggestion to further investigate potential limitations in real-time monitoring during various surgical stages [56].	[33,38,52,56]
Non-Invasive Health Monitoring	**Opportunity**	- Various studies, including Ha et al. [50] and Zhang et al. [66], discussed the use of electronic tattoos for continuous, noninvasive health monitoring, covering parameters like blood pressure and glucose levels.	[33,38,47,50,52,56,66]
Non-Invasive Health Monitoring	**Limitation**	- Challenges in achieving continuous, real-time monitoring of various health parameters [66]. - Potential limitations in the accuracy of blood pressure estimation [50].	[33,38,47,50,52,56,66]
Imaging and Multiplexed Detection	**Opportunity**	- Laroscelle et al. [46] explored the use of UV-excited luminescent tattoo inks for imaging in radiotherapy. - Piro et al. [67] discussed advancements in wearable skin chemical sensors for multiplexed detection.	[38,46,52,67]
Imaging and Multiplexed Detection	**Limitation**	- Challenges in achieving high-resolution imaging with luminescent tattoo inks [46]. - Potential limitations in multiplexed detection sensitivity [67].	[38,46,52,67]
Wearable Chemical Sensors	**Opportunity**	- Sempionatto et al. [45] explored wearable chemical sensors for noninvasive electrochemical detection of sweat vitamin C. - Gong et al. [59] developed highly sensitive, wearable strain sensors using polyaniline microparticles and gold nanowire films.	[38,45,52,59]
Wearable Chemical Sensors	**Limitation**	- Challenges related to the selective detection of specific biomarkers in sweat [45]. - Potential limitations in durability and stability of wearable chemical sensors [59].	[38,45,52,59]
Cost-Effective Fabrication Methods	**Opportunity**	- Wang et al. [62] and Bandodkar et al. [61] demonstrated cost-effective fabrication methods for wearable e-tattoo sensors and sodium sensors, respectively.	[38,52,61,62]
Cost-Effective Fabrication Methods	**Limitation**	- Challenges related to the scalability of cost-effective fabrication methods for large-scale production [62]. - Potential limitations in achieving high-performance sodium sensors [61].	[38,52,61,62]
Security Applications	**Opportunity**	- Laroscelle et al. [46] explored the use of luminescent tattoo inks as fiducial markers in radiotherapy, suggesting potential security applications in field verification during MV dose delivery. - Piro et al. [67] discussed sensing tattoos in security applications.	[38,46,52,67]
Security Applications	**Limitation**	- Challenges in achieving a significant signal-to-noise ratio improvement for security applications [46]. - Suggestion to deepen the investigation in the use of luminescent tattoo inks for real-time field verification during MV dose delivery [46].	[38,46,52,67]

## Supplementary Materials

The following supporting information can be downloaded at: https://www.mdpi.com/article/10.3390/bioengineering11040376/s1, Table S1: Key elements/points emerging on from the overview of the articles.; Table S2: Key elements/points emerging on the overview of reviews.; Box S1: The proposed composite keys.
