# Supplementary material for: Smart Tattoo Sensors 2.0: A Ten-Year Progress Report through a Narrative Review
Source: Bioengineering (Basel). 2024 Apr 13;11(4):376. doi: 10.3390/bioengineering11040376 (PMC11048663; doi:10.3390/bioengineering11040376)
Supplement: Supplementary file 1 [file bioengineering-11-00376-s001.zip › bioengineering-2868132-supplementary.pdf]

Supplementary material

Table S1 summarizes for the sake of clarity the key emerging points of the overviewed articles. Box S1 reports the composite keys.

Table S1. Key elements/points emerging from the overview of the articles.

Study	Key points
Minwoo et al. [32]	The study introduced affordable and user-friendly disposable wearable sensors using glossy paper and liquid bandages, featuring spray-coated silver nanowire composite films for stable attachment to human skin. The method enables continuous recording of electrophysiological signals like electromyogram (EMG), electrocardiogram (ECG), and electrooculogram (EOG).
Wang et al. [33]	The study contribution addressed noninvasive venous blood oxygenation monitoring with a soft wearable e-tattoo sensor measuring arterial and venous pulses from the wrist. The study proposes spatial filtering to mitigate crosstalk between arterial and venous signals, enhancing clinical diagnoses of conditions like sepsis and shock.
Lim et al. [34]	Authors focused on surface electromyography (sEMG) sensors, introducing an integrated system called an electronic tattoo (e-tattoo) that improves long-term comfort and demonstrates effectiveness in monitoring muscle activities with enhanced signal quality during active movements.
Vural et al. [35]	Authors conducted a perspective study on wearable devices for comprehensive health monitoring, emphasizing the importance of noninvasive electrochemical sensors in detecting biomarkers in various body fluids, covering recent articles and discussing implementation challenges and future prospects.
He et al. [37]	The work proposed a colorimetric dermal tattoo biosensor for multiplexed detection of health-related biomarkers in skin interstitial fluid, showing potential for simultaneous detection of multiple biomarkers in vitro, ex vivo, and in vivo.
Chen et al. [40]	Authors presented electronic tattoos as lightweight and noninvasive wearable electronics, addressing challenges in flexibility, skin biocompatibility, adhesion, repairability, and erasability by using a dynamic ionic liquid. The electronic tattoo demonstrated excellent sensing performance in response to temperature variation and tensile strain, monitoring body temperature, pulse, and movement.
Zhao et al. [41]	The scholars developed highly conductive graphene nanosheet film-based tattoo dry electrodes (TDEs) for human electrophysiology and strain sensing, exhibiting lower skin–electrode contact impedance and enabling accurate detection of electrocardiogram and electromyogram during 24-hour wearing.
Gogurla et al. [42]	Research utilized natural silk protein with carbon nanotubes to create an epidermal electronic tattoo (e-tattoo) system for multifunctional applications, integrating electrically and optically active heaters, a temperature sensor, a stimulator for drug delivery, and real-time electrophysiological signal detectors onto human skin.
Taccola et al.[43]	Authors developed ultra-conformable temporary tattoo electrodes (TTEs) for electrophysiological recordings, utilizing ink-jet printing of PEDOT:PSS on temporary tattoo paper for real-time monitoring of respiration through transthoracic impedance measurements.
Kedambaimole et al. [44]	Authors showcased a Ti3C2-MXene resistor as an ultrathin skin-mountable temporary tattoo for highly sensitive strain sensing, allowing inconspicuous monitoring of vital health parameters such as pulse rate, respiration rate, and surface electromyography.
Sempionatto et al. [45]	The proposal explored wearable chemical sensors for personalized nutrition solutions, presenting an epidermal biosensor for noninvasive electrochemical detection of sweat vitamin C. The biosensor demonstrated selective response, mechanical resiliency, and potential for personalized nutrition assessments.

Laroscelle et al. [46]	Authors explored the use of UV-excited luminescent tattoo inks as fiducial markers in radiotherapy patient alignment, demonstrating the feasibility of visualizing these inks under megavoltage radiation for real-time field verification during MV dose delivery.
Chen et al. [47]	Authors developed a transient epidermal sensor using water-soluble polyethylene oxide (PEO) substrate and poly(3,4-ethylenedioxythiophene) polystyrene sulfonate (PEDOT:PSS) conjugated polymer. The sensor exhibited stable electronic properties under static stress, dynamic load, and transient conditions, dissolving in water after use.
Kim et al. [48]	The study proposed a quantitative imaging method to monitor tattoo removal using laser treatment, accurately quantifying tattoo contrast variations post-laser treatment without thermal injury.
Ha et al. [50]	Scholars introduced a stretchable, ultrathin seismocardiography (SCG) sensing e-tattoo based on a 28 μm thick piezoelectric polymer, PVDF. The EMAC tattoo allowed synchronous ECG and SCG measurements, correlating systolic time interval (STI) with blood pressure and demonstrating reduced motion artifacts.
Kim et al [51]	The contribution presented a wearable epidermal platform for simultaneous, noninvasive sampling and analysis of sweat and interstitial fluid (ISF) using a single device, enabling real-time measurement of biomarkers in human subjects.
Park et al. [53]	Authors developed a cost-effective, large-area fabrication method for reduced graphene oxide (rGO) films on flexible polymer substrates, exhibiting compatibility with electronic module chips and flexible humidity sensors.
Stier et al. [54]	The study introduced a large-area, ultra-thin, ultra-soft tattoo-like heater with autonomous PID temperature control, demonstrating the ability to maintain a target temperature and conform to skin deformation for long-term wearability in medical applications.
Mishra et al. [55]	The contribution developed flexible epidermal tattoo and textile-based electrochemical biosensors for vapor-phase detection of organophosphorus nerve agents, offering rapid and selective square-wave voltametric detection of OP vapors.
Ameri et al. [57]	Authors presented graphene electronic tattoos (GETs) as sub-micrometer thick, multimodal sensors designed for wearability, demonstrating successful application in measuring various physiological parameters, including ECG, EMG, EEG, skin temperature, and hydration.
Jeong et al. [58]	Authors introduced a low-cost, wireless, stretchable biosensor integrating various sensors using a "cut-and-paste" method, demonstrating high-fidelity sensing for applications like skin thermography and photometry.
Gong et al. [59]	The proposal developed highly sensitive, wearable strain sensors using polyaniline microparticles and gold nanowire (AuNW) films, exhibiting enhanced conductivity and sensitivity, improved stretchability, water resistance, and direct interfacing with wireless circuitry.
Bandodkar et al. [60]	Authors demonstrated an all-printed temporary tattoo-based glucose sensor for noninvasive glycemic monitoring, exhibiting a linear response to glucose levels in human subjects.
Bandodkar et al. [61]	The same authors of the previous study developed an epidermal potentiometric sodium sensor embedded in a temporary-transfer tattoo for real-time monitoring of sodium in human perspiration, demonstrating continuous monitoring of sweat sodium dynamics during exercise.
Wang et al. [62]	This study explores the growing realm of wearable electronics, introducing a versatile e-tattoo with a mixed-dimensional matrix network. The e-tattoo's unique design offers exceptional sensing capabilities, envisioning it as a promising platform for the next generation of wearable electronics, overcoming challenges in skin conformity.
Jang et al. [63]	Tackling inconspicuous electrodermal activity (EDA) sensing, the study presents imperceptible graphene e-tattoos on the palm. Overcoming issues of obstructiveness, the integration of heterogeneous serpentine ribbons demonstrates effective

	ambulatory EDA monitoring in real-life conditions, validated against established gold standards.
Galliani [64]	Centered on wearable electronic device integration, this work introduces a method for crafting conformable thin sensors with cutaneous electrode patterning. Emphasizing high-quality recordings and long-term functionality, it calls for comparative studies against clinical gold standards, aiming to contribute to wearable sensors for human health monitoring.
Tang et al. [65]	Unveiling a groundbreaking electronic tattoo for health and movement sensing, the study highlights characteristics like high stretchability and conformality. The tattoo, transferable to various surfaces, simplifies fabrication with a layer-by-layer approach, showcasing practical applications like temperature adjustment, movement monitoring, and remote control of robots.

Table S2 reports the key points emerging from each review study.

Table S2. Key elements/points emerging on the overview of reviews.

Review Study	Key points
Makinia et al. [31]	Introduction of cost-effective, conformable skin piezoelectric sensors with organic electrochemical transistors (OECTs) for real-time electrophysiological signal monitoring, paving the way for fully printed wearable devices.
Manasa et al. [36].	Comprehensive review on the evolution of skin glucose monitoring devices, emphasizing real-time continuous glucose monitoring systems (rt-CGMs) and focusing on microneedle (MN) array systems. Discusses material design, assembly techniques, and envisions future developments in clinical applications.
Sharma et al. [38]	Exploration of nanostructured ion-selective membranes (ISMs) for biomedical applications, emphasizing miniaturization for implantable or wearable devices like smartwatches and tattoos. Critical review covers developments in ISM construction, addressing challenges and providing recommendations for improved accuracy.
Pazos et al. [39]	Investigation of traditional tattoo inks for health monitoring using optical biosensors, highlighting diagnostic capabilities. Emphasizes the need for further research on tattoo ink composition, related complications, and degradation kinetics. Discusses clinical advantages, challenges for in vivo implantation, and potential for self-controlling health management.
Yetisen et al.[49]	Introduction of minimally invasive, injectable dermal biosensors for monitoring pH, glucose, and albumin concentrations. Demonstrates multiplexing capabilities with potential applications in managing acid-base homeostasis, diabetes, and liver failure in point-of-care settings.
Valentini et al. [52]	Review of recent advancements in portable sensor technologies for Cultural Heritage, introducing a trend of portable tattoo devices for on-the-spot analysis. Crucial for immovable and intangible Cultural Heritage objects, proposing non-invasive, non-destructive portable contact sensors directly applied to art objects and surfaces.
Michard et al. [56]	Exploration of smartphones and electronic tablets in surgical care, identifying their potential from pre-rehabilitation to rehabilitation. Discusses digital applications, connected sensors (including tattoo based sensors), and post-surgery monitoring using smartphones. Calls for further studies to assess the impact of digital tools on surgical outcomes and postoperative complications.
Zhang et al. [66]	Highlight the exorbitant global cost of diabetes care, exceeding USD 1 trillion, emphasizing the need for innovation. Their review explores

	wearable glucose sensors, embedded in various platforms, offering non-invasive, real-time glucose analysis and advocating for advancements in materials and construction to enhance diabetes management.
Piro et al. [67]	Reports an extensive exploration of wearable skin chemical sensors addresses key applications with a focus on analyte detection and sensor performance. The review not only identifies existing gaps but strategically proposes future directions, aiming for the effective commercialization of these sensors and their transformative impact on healthcare.

Box S1. The proposed composite keys

" ((sensor[Title/Abstract]) OR (biosensor[Title/Abstract]) OR (biosensing[Title/Abstract]) OR (skin sensor[Title/Abstract])) AND (tattoo[Title/Abstract]) "
"((sensor[Title/Abstract]) OR (biosensor[Title/Abstract]) OR (biosensing[Title/Abstract]) OR (skin sensor[Title/Abstract])) AND (tattoo[Title/Abstract]) AND ((covid-19[Title/Abstract]) OR(SARS-CoV-2[Title/Abstract]))
"((sensor[Title/Abstract]) OR (biosensor[Title/Abstract]) OR (biosensing[Title/Abstract]) OR (skin sensor[Title/Abstract])) AND (tattoo[Title/Abstract]) AND (Medical Device[Title/Abstract])"
"((sensor[Title/Abstract]) OR (biosensor[Title/Abstract]) OR (biosensing[Title/Abstract]) OR (skin sensor[Title/Abstract])) AND (tattoo[Title/Abstract]) AND (regulation [Title/Abstract])"
"((sensor[Title/Abstract]) OR (biosensor[Title/Abstract]) OR (biosensing[Title/Abstract]) OR (skin sensor[Title/Abstract])) AND (tattoo[Title/Abstract]) AND (cybersecurity [Title/Abstract])"
